# The PK/PD Integration and Resistance of Tilmicosin against *Mycoplasma hyopneumoniae*

**DOI:** 10.3390/pathogens9060487

**Published:** 2020-06-19

**Authors:** Zilong Huang, Zixuan Hu, Haorui Zheng, Xirui Xia, Xiaoyan Gu, Xiangguang Shen, Hong Yang, Huanzhong Ding

**Affiliations:** 1 Guangdong Key Laboratory for Veterinary Drug Development and Safety Evaluation, South China Agriculture University, 483 Wushan Road, Guangzhou 510642, China; huangzilong@stu.scau.edu.cn (Z.H.); huzixuan@stu.scau.edu.cn (Z.H.); haoruizheng@stu.scau.edu.cn (H.Z.); xiaxirui@stu.scau.edu.cn (X.X.); guxianyan@scau.edu.cn (X.G.); shenxg@scau.edu.cn (X.S.); 2 School of life science and engineering, Foshan University, Foshan 510642, China; yhong007@fosu.edu.cn

**Keywords:** *Mycoplasma hyopneumoniae*, tilmicosin, in vitro dynamic model, pharmacokinetic, pharmacodynamic, enzootic pneumonia, resistance

## Abstract

*Mycoplasma hyopneumoniae* is the major pathogen causing enzootic pneumonia in pigs. *M. hyopneumoniae* infection can lead to considerable economic losses in the pig-breeding industry. Here, this study established a first-order absorption, one-compartment model to study the relationship between the pharmacokinetics/pharmacodynamics (PK/PD) index of tilmicosin against *M. hyopneumoniae* in vitro. We simulated different drug concentrations of timicosin in the fluid lining the lung epithelia of pigs. The minimum inhibitory concentration (MIC) of tilmicosin against *M. hyopneumoniae* with an inoculum of 10^6^ CFU/mL was 1.6 μg/mL using the microdilution method. Static time–kill curves showed that if the drug concentration >1 MIC, the antibacterial effect showed different degrees of inhibition. At 32 MIC, the amount of bacteria decreased by 3.16 log_10_ CFU/mL, thereby achieving a mycoplasmacidal effect. The *M. hyopneumoniae* count was reduced from 3.61 to 5.11 log_10_ CFU/mL upon incubation for 96 h in a dynamic model with a dose of 40–200 mg, thereby achieving mycoplasmacidal activity. The area under the concentration-time curve over 96 h divided by the MIC (AUC_0–96 h_/MIC) was the best-fit PK/PD parameters for predicting the antibacterial activity of tilmicosin against *M. hyopneumoniae* (*R*^2^ = 0.99), suggesting that tilmicosin had concentration-dependent activity. The estimated value for AUC_0–96 h_/MIC for 2log_10_ (CFU/mL) reduction and 3log_10_ (CFU/mL) reduction from baseline was 70.55 h and 96.72 h. Four *M. hyopneumoniae* strains (M1–M4) with reduced sensitivity to tilmicosin were isolated from the four dose groups. The susceptibility of these strains to tylosin, erythromycin and lincomycin was also reduced significantly. For sequencing analyses of 23S rRNA, an acquired A2058G transition in region V was found only in resistant *M. hyopneumoniae* strains (M3, M4). In conclusion, in an in vitro model, the effect of tilmicosin against *M. hyopneumoniae* was concentration-dependent and had a therapeutic effect. These results will help to design the optimal dosing regimen for tilmicosin in *M. hyopneumoniae* infection, and minimize the emergence of resistant bacteria.

## 1. Introduction

*Mycoplasma hyopneumoniae* is the primary pathogen of mycoplasmal pneumonia in pigs. *M. hyopneumoniae* is widespread in various regions, and can cause huge economic losses to the pig industry [1]. Infected pigs are the main source of infection. The pathogen can be transmitted directly through air and contact, so the infection rate is extremely high [2]. Once flocks of pigs are infected, eradication is difficult because *M. hyopneumoniae* can transmit vertically [3,4].

Antibiotic treatment is one of the main methods for controlling *M. hyopneumoniae* [5]. Tilmicosin is a broad-spectrum, macrolide antibiotic used commonly in animals, especially against *Mycoplasma* species [6]. It has a long elimination half-life and high concentration in lung tissues [7,8]. The unique antibacterial mechanism of tilmicosin is perfect for the treatment of *M. hyopneumoniae* infections. It is combined with the 50S large subunit of bacterial ribosomes to exert an antibacterial effect, rather than acting on the cell wall. However, the unreasonable use and abuse of macrolides has led to the emergence of drug resistance [9,10]. Most of the resistance mechanisms to macrolides include active efflux mechanisms, i.e., changes in target molecules bound by drugs [11]. Resistance to macrolides is associated with mutations in domains II or V of 23S rRNA genes, or the rplD and rplV genes encoding ribosomal proteins L4 and L22 [10,12,13].

Due to the difficulty of culturing and counting *M. hyopneumoniae*, the pharmacokinetic/pharmacodynamic (PK/PD) profiles of tilmicosin against *M. hyopneumoniae* are very limited. Also, establishing an infection model of *M. hyopneumoniae* in vivo is challenging. Therefore, it is a feasible to establish an in vitro dynamic model to evaluate the effect of tilmicosin against *M. hyopneumoniae*. In vitro PK/PD models have been used widely to optimize dose regimens, monitor antimicrobial activity and prevent the emergence of resistant bacteria [14]. Such models can simulate changes in drug concentration in animals, but also eliminate differences among animals [15]. Moreover, the PK/PD parameters in the in vitro model are very similar to those in the animal-infection model [16,17,18]. Therefore, establishing an in vitro dynamic model appears to be a viable way to evaluate the effects of tilmicosin on *M. hyopneumoniae*.

This study wished to apply the one-compartment infection model in vitro to determine the PK/PD indices of tilmicosin against *M. hyopneumoniae*; in this way, we could investigate the mechanism of resistance. This model can be used as a reference to optimize the dosing regimen for tilmicosin against *M. hyopneumoniae*.

## 2. Materials and Methods

### 2.1. Materials

A standard strain of *M. hyopneumoniae* (ATCC 25934) was obtained from the Chinese Veterinary Microorganism Culture Collection Center (Beijing, China) and stored at −80 °C. Tilmicosin (75.8%), tylosin (82.6%), erythromycin (85.0%), tiamulin (99.0%), doxycycline (85.8%) and enrofloxacin (99.0%) were kindly supplied by Guangdong Dahuanong Animal Health Products (Xincheng, China). Amikacin (99.0%) and lincomycin (84.6%) were purchased from Guangdong Puboxing Animal Health Products (Guangzhou, China) and stored at −80 °C before use.

A fresh stock solution (1280 mg/L) of each antibacterial agent was prepared for each experiment. Broth medium base was purchased from Qingdao Hope Biological Technology (Qingdao, China). The reduced form of nicotinamide adenine dinucleotide was obtained from Beijing Newprobe Biotechnology (Beijing, China). L-Cysteine was purchased from Beijing Solarbio Science and Technology (Beijing, China).

### 2.2. Determination of the Minimum Inhibitory Concentration (MIC)

The MIC of tilmicosin against *M. hyopneumoniae* was determined by a modified version of the MIC assay, as described by Tanner and Wu [19]. Briefly, dilutions of exponential-phase cells at 10^5^, 10^6^ and 10^7^ CFU/mL (100 μL) were added to an equal volume of drug-containing medium in each well in a 96-well plate. The tilmicosin concentration in the 96-well plate was 0.05–12.8 μg/mL. A growth control (lacking antibiotic), sterility control (sterile broth at pH 7.7) and endpoint control (blank medium at pH 6.5) were included. Plates were incubated at 37 °C in a humidified atmosphere of 5% CO_2_ until the growth group and endpoint control were the same color. The MIC was defined as the minimal concentration of antibacterial agent that resulted in no color change. All experiments were carried out in triplicate.

According to the method described by Hannan et al. [20], plates containing a series of tilmicosin concentrations (1.6–25.6 μg/mL) were prepared. Samples (10 μL) of cultures with an inoculum of 10^5^, 10^6^ and 10^7^ CFU/mL were also applied to the drug plates. A blank-growth control group was also used and comprised cells spread on plates lacking the drug. Plates were incubated for ≥7 days. The lowest concentration without *M. hyopneumoniae* growth was determined as the MIC. All experiments were carried out in triplicate.

### 2.3. Time–Kill Curves

Four milliliters of blank medium, 0.5 mL of 10-times the final drug concentration, and 0.5 mL of logarithmic *M. hyopneumoniae* were added to a bottle in turn and then mixed. The tilmicosin concentration in the culture system was in a certain range (1/2, 1, 2, 4, 8, 16, and 32 -times the MIC that was determined for an *M. hyopneumoniae* inoculum of 10^6^ CFU/mL). A growth control (not exposed to the drug) and a sterility control (medium at pH 7.7 without the drug or *M. hyopneumoniae*) were indispensable. Penicillin bottles were cultured for 60 h in the environments described above. Aliquots (100 μL) of the culture were taken from each bottle at 0, 1, 3, 6, 9, 12, 24, 36, 48 and 60 h to detect the *M. hyopneumoniae* population. After 7 days, the results were read using an inverted microscope (Leica, Weztlar, Germany).

### 2.4. PK/PD Model In Vitro and Dosing Regimens

**Figure 1:**
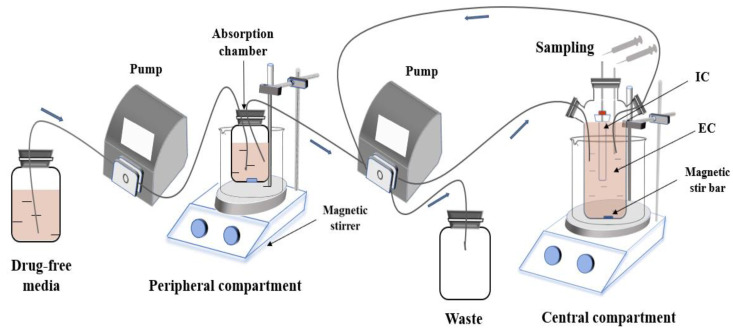
An in vitro model that simulates the pharmacokinetics of tilmicosin in the fluid lining the lung epithelia of pigs and determines the effects of tilmicosin on the growth and susceptibility of *M. hyopneumoniae.* EC, external compartment; IC, internal compartment.

This study used a previously described in vitro dynamic model [21]. This experiment was done to simulate the timicosin concentration in the fluid lining the lung epithelia of pigs [8]. The model was applied according to the outline shown in Figure 1.

Briefly, the model system consists of three parts: the first is an absorption chamber containing a drug medium as a site of administration; the second is a central chamber which comprises 300 mL of sterile medium (external compartment (EC)) and a 10-mL dialysis tube (internal compartment (IC)); the third part is a reserve room for fresh media. At the same time, the waste liquid is collected in the waste liquid chamber.

In this experiment, by referring to the pharmacokinetic data obtained from the in vivo pharmacokinetic experiments of tilmicosin, a fitting analysis was performed to obtain the required pharmacokinetic parameters and set the flow rate of the pump. Then, the pump model was used to simulate the absorption and elimination process of tilmicosin. Based on this, the relationship between the pharmacokinetic and pharmacodynamic effects of different doses of tilmicosin against *M. hyopneumoniae* was studied. The hollow fiber systems have cartridges containing hundreds of hollow fibers which separate the bacteria in the peripheral compartment from the central compartment. The large number of hollow fibers provide a large surface area for drugs to readily diffuse between the central reservoir and the peripheral compartment.

The model parameters were determined by the colonization site of *M. hyopneumoniae* and PK characteristics of tilmicosin in pigs. The parameter values of absorption half-life, elimination half-life and flow rate of peristaltic pumps were 12.17 h, 17.16 h and 0.29 mL/min, respectively. The dosage regimen for this experiment was based on the clinically recommended dosage of tilmicosin and several doses above and below it. The antibacterial effect of tilmicosin was simulated and observed at, and above or below, the recommended dose against *M. hyopneumoniae*. Eight dose groups (10, 20, 40, 60, 80, 120, 160 and 200 mg) were designed for the in vitro dynamic model. Increasing the speed of the magnetic stirrer as the drug was injected into the absorption chamber achieved a rapid balance between the inside and outside of the dialysis membrane simultaneously.

Samples (2 mL) were collected from the EC 1, 3, 6, 9, 12, 24, 36, 48, 72 and 96 h after administration, and then stored at −20 °C until analyses. Samples (100 μL) were taken from the IC before dosing, as well as 6, 12, 24, 36, 48, 72 and 96 h after administration. The collected samples were used to detect the number of, and susceptibility to, *M. hyopneumoniae*.

### 2.5. Determination of the Tilmicosin Concentration in the Medium

The tilmicosin concentration in the medium was analyzed using a high-performance liquid chromatography unit (1200 series; Agilent Technologies, Santa Clara, CA, USA) and a triple quadrupole mass spectrometer (6410; Agilent Technologies, Santa Clara, CA, USA) equipped with an electrospray ionization source. The analytical method used was as described by Huang et al. [18]. The standard curve (*R*^2^ > 0.99) was defined by six calibration standards of tilmicosin, with a final concentration ranging from 5 ng/mL to 500 ng/mL. The limit of detection (LoD) and limit of quantification (LoQ) in the medium were 0.5 ng/mL and 1 ng/mL, respectively.

### 2.6. Integration and Modeling of PK/PD

Three important PK/PD indices were calculated by integrating PK parameters and MIC values in vitro: the peak concentration by MIC (C_max_/MIC), the area under the concentration–time curve over 96 h divided by the MIC (AUC_0–96 h_/MIC) and the cumulative time at which the concentration exceeds the MIC (%T > MIC).

The correlation between the PK/PD indices and antimicrobial activity against *M. hyopneumoniae* was analyzed using WinNonlin (Certara, Princeton, NJ, USA). We chose the inhibitory sigmoid *E*_max_ model to analyze data:

$$E\;=\;E_{\max}\;-\;\frac{(E_{\max}\;-\;E_{0})\;\times \;C_{e}^{N}}{{EC}_{50}^{N}\;+\;C_{e}^{N}}$$

where *E* is the anti- *M. hyopneumoniae* effect, *E*_max_ is the change in the amount of *M. hyopneumoniae* in the control group at a 96-h interval, *E*_0_ is the largest anti- *M. hyopneumoniae* effect, determined as log_10_CFU/mL reduction at the same interval, *C_e_* represents the PK/PD indices (%T > MIC, C_max_/MIC and AUC_0–96 h_/MIC), *N* is the Hill coefficient that describes the steepness of the PK/PD indices–effect curve, and *EC*_50_ is the corresponding PK/PD value when the anti- *M. hyopneumoniae* effect reaches 50% of the maximum antibacterial effect. *R*^2^ was calculated for each assay.

### 2.7. Susceptibility Testing of M. hyopneumoniae and DNA Sequencing

This study used the method described by Huang et al. [18] with slight modifications. Briefly, 100 μL of the bacteria was collected at the final time point in the IC qas. Then, every 10 μL of the bacterial solution was placed on the surface of the drug plate containing 1 × MIC concentration. After 7 days of culture, colonies that resumed growth were transferred to blank liquid medium and subcultured five times until their growth was stable. The MIC of these strains was redetermined, and colonies with reduced sensitivity to tilmicosin were screened. The selected drug-resistant bacteria were named M1, M2, M3 and M4. After five generations, amplification by polymerase chain reaction and sequencing of stabilized MIC mutants was carried out using Sanger sequencing by TsingKe Biological Technology (Chengdu, China). The sensitivity of these strains to other antimicrobial agents (lincomycin, amikacin, enrofloxacin, doxycycline, tiamulin, erythromycin, and tylosin) was also tested.

## 3. Results

### 3.1. Susceptibility Determination

The MIC of tilmicosin against *M. hyopneumoniae* with inoculums of 10^5^, 10^6^, and 10^7^ CFU/mL was 0.8, 1.6 and 1.6 μg/mL using the microdilution method, and 3.2, 6.4 and 6.4 μg/mL using the agar dilution method, respectively.

### 3.2. Analyses of Time–Kill Curves

**Figure 2:**
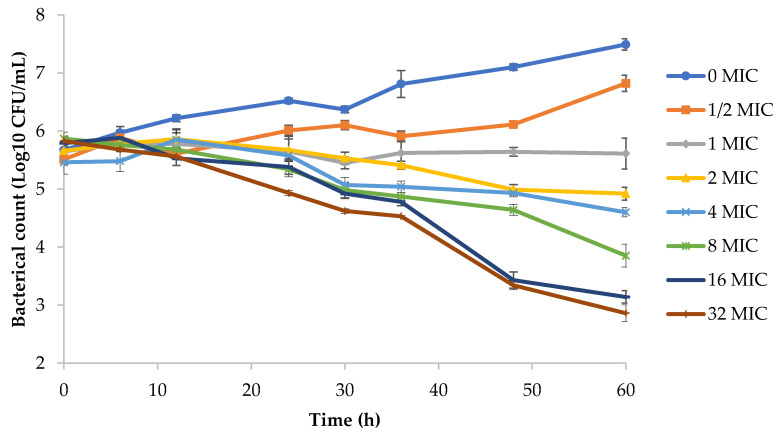
Time–kill studies of tilmicosin against *M. hyopneumoniae* at constant concentrations. MIC, minimum inhibitory concentration; CFU, colony-forming units. Data points represent the geometric mean values of three experiments.

The in vitro static bactericidal curves of different concentrations of tilmicosin against *M. hyopneumoniae* are shown in Figure 2. When the drug concentration was 0.5 MIC, the bacteria continued to grow, and the number of bacteria increased by 1.31 log_10_ CFU/mL. The number of bacteria in the blank-growth control group increased by 1.82 log_10_ CFU/mL. When the drug concentration was >1 MIC, the antibacterial effect showed different degrees of inhibition. The bacteria at 2, 4, 8 and 16 MIC decreased by 0.742, 0.858, 2.03 and 2.65 log_10_ CFU/mL, respectively. At 32 MIC, the amount of bacteria decreased by 3.16 log_10_ CFU/mL, thereby achieving a mycoplasmacidal effect. In summary, the antibacterial effect of tilmicosin against *M. hyopneumoniae* was more obvious with an increase in the drug concentration.

**Figure 3:**
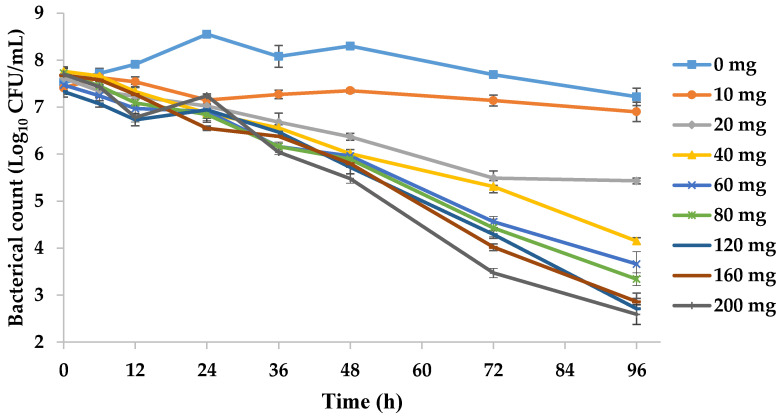
Dynamic time–kill curves were plotted at eight doses of tilmicosin. Data points represent the geometric mean values of three experiments.

The bactericidal curve of tilmicosin from the in vitro dynamic model at different clinically recommended doses is shown in Figure 3. Within 0 to 36 h, the number of bacteria under different drug doses did not decrease significantly, which suggested that the antibacterial effect of tilmicosin against *M. hyopneumoniae* took a long time to occur. With an increase in drug dose, tilmicosin showed increased activity against *M. hyopneumoniae*. The *M. hyopneumoniae* count was reduced from 3.61 to 5.11 log_10_ CFU/mL upon incubation for 96 h in a dynamic model with a dose of 40–200 mg, and achieved mycoplasmacidal activity.

### 3.3. PK in the In Vitro Dynamic Model

**Figure 4:**
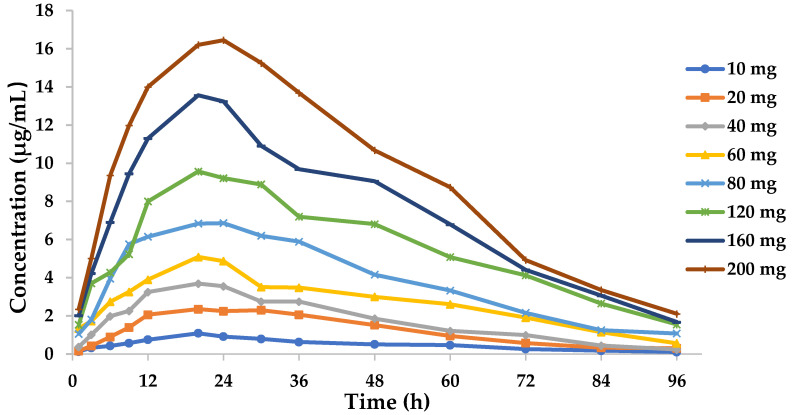
Concentration–time curves of eight doses of tilmicosin in the in vitro dynamic model.

The time–concentration curves for the in vitro dynamic model are shown in Figure 4. The PK parameters are summarized in Table 1. A standard curve was constructed by the addition of a specific concentration of tilmicosin to the drug-free medium at concentrations ranging from 0.005 μg/mL to 0.5 μg/mL (*R*^2^ > 0.99). The relative errors of elimination half-life and absorption half-life were −5.6 and 9.28%, respectively, both of which were within the normal range of ±15%. The LoD and LoQ for the developed method were 0.5 ng/mL and 1 ng/mL, respectively.

### 3.4. Modeling and Analyses of PK/PD

**Figure 5:**
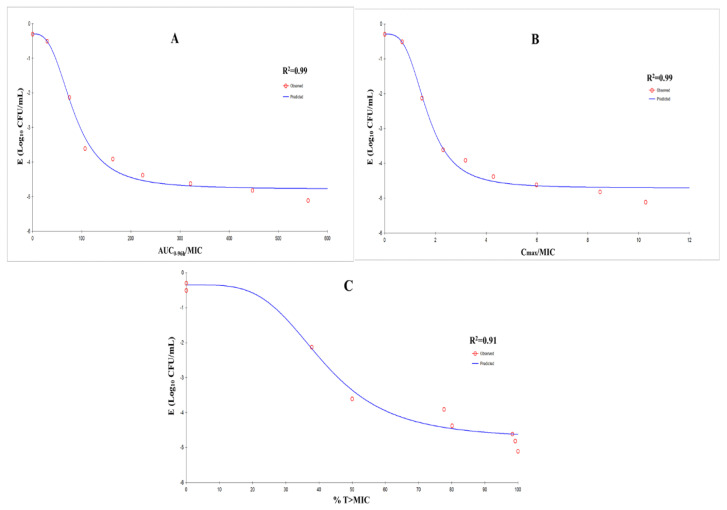
*E*_max_ relationships for three PK–PD parameters versus the antimycoplasmal effect using curves. (**A**) AUC_0–96 h_/MIC–antimycoplasmal effect; (**B**) C_max_/MIC–antimycoplasmal effect; (**C**) %T > MIC–antimycoplasmal effect. *R*^2^ is the correlation coefficient; *E*_max_ is the sigmoid maximum effect; PK, pharmacokinetic; PD, pharmacodynamic; AUC, area under the concentration–time curve; MIC, minimum inhibitory concentration; %T > MIC is the cumulative time that the concentration exceeds the MIC; C_max_/MIC is the peak concentration by MIC.

The relationships between antibacterial effect and the PK/PD parameters of this dynamic model are shown in Figure 5. According to a simulation of the inhibitory sigmoid *E*_max_ model, the correlation coefficient between AUC_0−96 h_/MIC, %T > MIC, C_max_/MIC and the antibacterial effect was 0.99, 0.91 and 0.99, respectively. C_max_/MIC and AUC_0–96 h_/MIC were the best-fit PK/PD parameters for predicting the antibacterial activity of tilmicosin against *M. hyopneumoniae*, which suggested that tilmicosin had concentration-dependent activity. The estimated values for C_max_/MIC and AUC_0–96 h_/MIC for 2log_10_ (CFU/mL) reduction and 3log_10_ (CFU/mL) reduction from baseline were 1.44 and 1.91, and 70.55 h and 96.72 h, respectively, during a 96-h treatment period with tilmicosin. The obtained parameters of *E*_0_, *E*_max_ and *EC*_50_, and the Hill coefficient, are listed in Table 2.

### 3.5. Susceptibility Testing and Mutation Analyses

Four *M. hyopneumoniae* strains (M1, M2, M3, and M4) with reduced sensitivity to tilmicosin were isolated from the four dose groups (10, 60, 80, and 120 mg). The MIC of these strains to tilmicosin ranged from 25.6 μg/mL to 1638.4 μg/mL. Moreover, the MIC of strains M3 and M4 was significantly higher than that of the standard strains. Table 3 shows the changes in sensitivity of these strains to eight antimicrobial agents. The susceptibility of these strains to tylosin, erythromycin and lincomycin was also reduced significantly. For sequencing analyses of 23S rRNA (Table 4), we measured the gene sequences of regions V, L4 and L22, and compared them with standard strains. An acquired A2058G transition in region V was found only in the resistant *M. hyopneumoniae* strains (M3, M4), and a resistance mutation was not found in L4 or L22.

## 4. Discussion

Mycoplasmal pneumonia caused by *M. hyopneumoniae* infection is a major, worldwide problem in the pig industry. If *M. hyopneumoniae* is mixed with other pathogenic microorganisms, porcine respiratory disease complex can occur [1,22].

The rational use of antibiotics is the main measure to prevent and treat mycoplasmal pneumonia. However, *M. hyopneumoniae* presents technical difficulties in culturing and agar-plate enumeration, both in the culture environment and in the addition of nutrients [23]. *M. hyopneumoniae* grows slowly, and is inhibited competitively by other bacteria, so the isolation of *M. hyopneumoniae* strains is difficult [24]. Hence, studies on the PK/PD interaction of tilmicosin against *M. hyopneumoniae* are scarce.

The in vitro dynamic model has been used widely to study PK/PD interactions. Tam et al. [25] investigated the PK/PD relationship of polymyxin B against *Pseudomonas aeruginosa* using an in vitro dynamic model. They showed that polymyxin B had rapid and concentration-dependent bactericidal activity against *P. aeruginosa*, and that the effect was diminished if the amount of bacteria was increased. Also, study of the effect of doxycycline against *Mycoplasma gallisepticum* in an in vitro model led to the determination of the optimal PK/PD parameters to prevent drug resistance [26].

Here, this study reported on an in vitro PK/PD model of *M. hyopneumoniae* which simulated the PK of tilmicosin in bronchoalveolar lavage fluid after a single oral dose. The advantage of this model is that when it is difficult to establish an animal-infection model, the interaction between the drug and bacteria can be elucidated, and the change in drug sensitivity of the pathogen can be observed to study the drug-resistance mechanism.

Vicca et al. [27] determined the in vitro susceptibility of *M. hyopneumoniae* field isolates by a broth microdilution method. They showed that the MIC range of tilmicosin against *M. hyopneumoniae* was 0.25–16 μg/mL. Felde and colleagues reported that the MIC of tilmicosin against *M. hyopneumoniae* was 0.25–64 μg/mL [28]. Compared with those two studies, the MIC determined in the present study was within a reasonable range. The recommended turbidity of MIC testing against veterinary *Mycoplasma* species is 10^3^–10^5^ CFU/mL [29]. However, the turbidity of the MIC testing used in our experiments was 10^5^–10^7^ CFU/mL, and the inoculum used in the in vitro dynamic model experiment was high (10^7^ CFU/mL).

There were three main reasons for choosing a high inoculum. First, turbidity has little effect on the growth of *Mycoplasma* species or MIC determination [30]. Second, tilmicosin accumulates mainly in the lungs, so the concentration in lung tissues is much higher than in plasma. If the drug concentration in the lungs is simulated, low bacterial counts are eliminated rapidly. Third, mutant subpopulations are present at low frequencies (10^−6^ to 10^−8^) [31]. Therefore, a high inoculum may increase the likelihood of monitoring mutant strains and resistance mechanisms. The binding rates of plasma proteins were not considered in the in vitro dynamic assay, also because the tilmicosin concentration in plasma was much lower than that in the lungs. Using the drug concentration in plasma as a reference is not reasonable.

The time–kill curves in Figure 2 showed that the antibacterial effect was more obvious when increasing the drug concentration (1–32 × MIC). The same situation appeared in the in vitro dynamic time–kill curve (Figure 3). When the tilmicosin dose reached 40 mg, the bacteria decreased by 3.6 log_10_ CFU/mL. It is worth noting that the number of bacteria was slightly reduced in the blank-growth control group and low-dose group (10 mg). There are two reasons for this result. First, the nutritional conditions required for *M. hyopneumoniae* growth were compromised slightly due to the long period of the dynamic model test. Second, in the low-dose group, there was a postantibiotic sub-MIC effect (PA-SME), which may have slightly reduced the amount of bacteria.

The results of *E*_max_ model-fitting confirmed that the effect of tilmicosin on *M. hyopneumoniae* was concentration-dependent, and the parameters AUC_96h_/MIC and C_max_/MIC showed a strong correlation with antibacterial effects (*R*^2^ = 0.99). Most studies have shown that the %T > MIC parameter of macrolides is significantly associated with antimicrobial activity [32,33]. However, the antibacterial activity of azithromycin with a long elimination half-life is related to the AUC_24 h_/MIC parameter [34]. Therefore, the antibacterial activity of antibiotics is not static; it is dependent upon the characteristics of drugs and bacteria. The same antibiotic has different types of action on different bacteria [35]. A number of studies have shown that when macrolide antibiotics are concentration-dependent, the best PK/PD parameter is AUC/MIC [36,37,38,39]. The main reasons for the high correlation between AUC_96h_/MIC and C_max_/MIC in this experiment are the lack of fitting analysis data and the relatively close trend. Finally, it was decided that AUC_96h_/MIC should be used as the optimal parameter.

The study screened resistant strains of *M. hyopneumoniae* using drug-containing agar plates. Four strains were obtained with significantly reduced sensitivity to tilmicosin. Studies on the mechanism of resistance of *Mycoplasma* species to macrolides are limited to mutations in drug-target molecules and the efflux of antibacterial-active substances. It was found that A2058G mutation in domain V of the 23S rRNA gene (M3 and M4) was associated with resistance. No resistance-related differences were found in the ribosomal proteins L4 and L22. Studies have also shown that macrolide-resistant *M. hyopneumoniae* strains isolated from animals have mutations at positions 2057, 2058, 2059 or 2064 in the 23S rRNA gene [9,10].

This study had two main limitations. First, all experiments were undertaken in vitro. Although the effects of the drug on bacteria were studied carefully and thoroughly, the effects of the immune system of an animal on microorganisms were not considered. Second, only one standard strain of *M. hyopneumoniae* was tested, and testing clinical isolates is necessary to confirm our findings.

## 5. Conclusions

This was the first study on the PK/PD relationship of tilmicosin against *M. hyopneumoniae*. In the in vitro dynamic model, tilmicosin produced a maximal anti- *M. hyopneumoniae* effect of a 5.11 log_10_ (CFU/mL) reduction. The antibacterial effect of tilmicosin was concentration-dependent, and the best-fit PK/PD parameters were the AUC_0–96 h_/MIC (*R*^2^ = 0.99). The estimated values for AUC_0–96 h_/MIC for 2log_10_ (CFU/mL) reduction and 3log_10_ (CFU/mL) reduction from baseline were 70.55 h and 96.72 h. The A2058G mutation in region V of the 23S rRNA gene was found in the M3 and M4 strains. These results provide a reliable reference for animal experiments in vivo, and may help in the design of more rational treatments for *M. hyopneumoniae* infection.

## Figures and Tables

**Table 1:** Main pharmacokinetic parameters of different tilmicosin doses in the in vitro PK/PD model.

Pharmacokinetic Parameter	Dose Group (mg)								$\bar{X}$	Relative Deviation
	10	20	40	60	80	120	160	200		
t_1/2_Ka (h)	12.7	14.3	12.6	14.3	12.1	13.1	12.4	14.6	13.3	9.28%
t_1/2_Kel (h)	16.5	14.4	12.7	15.6	17.8	19.7	17.7	14.8	16.2	−5.6%
T_max_ (h)	20.8	20.7	18.2	21.6	21.9	23.1	21.3	21.2	21.1	1.3%
C_max_ (μg/mL)	1.09	2.35	3.69	5.09	6.86	9.57	13.6	16.5	——	——
AUC (h.mg/L)	47.2	119.6	190.4	260.5	357.9	513.9	715.4	896.6	——	——
*R* ^2^	0.963	0.986	0.939	0.963	0.918	0.975	0.988	0.997	——	——

Relative deviation = (PK parameters in vitro/PK parameters in vivo − 1) × 100%; —— no data.

**Table 2:** Estimation of PK/PD parameters (data are derived from the *E*_max_ model).

PK/PD Parameter	*E*_max_ (log_10_ CFU/mL)	*EC*_50_	*E*_0_ (log_10_ CFU/mL)	Hill’s Slope	*R* ^2^
AUC_0–96 h_/MIC (h)	−0.30	91.93	−5.28	1.65	0.99
C_max_/MIC	−0.30	1.80	−5.17	1.89	0.99

**Table 3:** MIC of six antimicrobial agents against *M. hyopneumoniae* (Mhp) and M1–M4 strains.

Strain	MIC Value of Antibiotics (μg/mL)							
	Tilmicosin	Tylosin	Erythromycin	Tiamulin	Doxycyclin	Enrofloxacin	Amikacin	Lincomycin
Mhp	1.6	0.0625	0.64	0.08	0.0625	0.025	1.6	0.125
M1	25.6	0.125	>40.96	0.08	0.0625	0.025	1.6	0.125
M2	819.2	8	>40.96	0.08	0.0625	0.025	1.6	64
M3	1638.4	16	>40.96	0.08	0.0625	0.025	1.6	256
M4	1638.4	16	>40.96	0.16	0.0625	0.025	1.6	256

M1 (10 mg), M2 (60 mg), M3 (80 mg) and M4 (120 mg) strains were selected from seven doses, respectively.

**Table 4:** Tilmicosin susceptibility and identification of resistant mutants associated with different doses of tilmicosin.

Dose (mg)	Strain	MIC (μg/mL)	23S rRNA		
			V Region	L4	L22
0	Mhp	1.6	——	——	——
80	M3	819.2	A2058G	——	——
120	M4	819.2	A2058G	——	——

–: No mutant was found; M3, M4: mutants were selected from a dose of 80 mg, and 120 mg, respectively.

## References

[1] Maes D., Segales J., Meyns T., Sibila M., Pieters M., Haesebrouck F. (2008). Control of Mycoplasma hyopneumoniae infections in pigs. Vet. Microbiol..

[2] Otake S., Dee S., Corzo C., Oliveira S., Deen J. (2010). Long-distance airborne transport of infectious PRRSV and Mycoplasma hyopneumoniae from a swine population infected with multiple viral variants. Vet. Microbiol..

[3] Sibila M., Nofrarias M., Lopez-Soria S., Segales J., Riera P., Llopart D., Calsamiglia M. (2007). Exploratory field study on Mycoplasma hyopneumoniae infection in suckling pigs. Vet. Microbiol..

[4] Nathues H., Doehring S., Woeste H., Fahrion A.S., Doherr M.G., Beilage E.G. (2013). Individual risk factors for Mycoplasma hyopneumoniae infections in suckling pigs at the age of weaning. Acta Vet. Scand..

[5] van den Bogaard A.E. (1988). The importance of laboratory data for a rational antimicrobial therapy in veterinary practice. Tijdschr. Diergeneeskd..

[6] Ziv G., Shem-Tov M., Glickman A., Winkler M., Saran A. (1995). Tilmicosin antibacterial activity and pharmacokinetics in cows. J. Vet. Pharmacol. Ther..

[7] Shen J.Z., Li C., Jiang H.Y., Zhang S.X., Guo P., Ding S.Y., Li X.W. (2005). Pharmacokinetics of tilmicosin after oral administration in swine. Am. J. Vet. Res..

[8] Zhang P., Hao H.H., Li J., Ahmad I., Cheng G.Y., Chen D.M., Tao Y.F., Huang L.L., Wang Y.L., Dai M.H. (2016). The Epidemiologic and Pharmacodynamic Cutoff Values of Tilmicosin against Haemophilus parasuis. Front. Microbiol..

[9] Stakenborg T., Vicca J., Butaye P., Maes D., Minion F.C., Peeters J., De Kruif A., Haesebrouck F. (2005). Characterization of in vivo acquired resistance of Mycoplasma hyopneumoniae to macrolides and lincosamides. Microb. Drug Resist..

[10] Qiu G., Rui Y.P., Zhang J.L., Zhang L.H., Huang S.C., Wu Q.X., Li K., Han Z.Q., Liu S.Z., Li J.K. (2018). Macrolide-Resistance Selection in Tibetan Pigs with a High Load of Mycoplasma hyopneumoniae. Microb. Drug Resist..

[11] Weisblum B. (1995). Erythromycin resistance by ribosome modification. Antimicrob. Agents Chemother..

[12] Gerchman I. (2011). Characterization of in vivo-acquired resistance to macrolides of Mycoplasma gallisepticum strains isolated from poultry. Vet. Res..

[13] Vester B., Douthwaite S. (2001). Macrolide resistance conferred by base substitutions in 23S rRNA. Antimicrob. Agents Chemother..

[14] Vinks A.A., Derendorf H., Mouton J.W. (2014). Fundamentals of Antimicrobial Pharmacokinetics and Pharmacodynamics.

[15] Liang W., Chen Y.C., Cao Y.R., Liu X.F., Huang J., Hu J.L., Zhao M., Guo Q.L., Zhang S.J., Wu X.J. (2013). Pharmacokinetics and Pharmacodynamics of Nemonoxacin against Streptococcus pneumoniae in an In Vitro Infection Model. Antimicrob. Agents Chemother..

[16] Andes D., Craig W.A. (2002). Pharmacodynamics of the new fluoroquinolone gatifloxacin in murine thigh and lung infection models. Antimicrob. Agents Chemother..

[17] Booker B.M., Smith P.F., Forrest A., Bullock J., Kelchlin P., Bhavnani S.M., Jones R.N., Ambrose P.G. (2005). Application of an in vitro infection model and simulation for reevaluation of fluoroquinolone breakpoints for Salmonella enterica serotype Typhi. Antimicrob. Agents Chemother..

[18] Huang Z.L., Wu Y.Z., Zhou Z.C., Xia X.R., Gu X.Y., Cai Q.R., Shen X.G., Yang H., Ding H.Z. (2019). Pharmacokinetic and Pharmacodynamic Integration and Resistance Analysis of Tilmicosin Against Mycoplasma gallisepticum in an In Vitro Dynamic Model. Front. Pharmacol..

[19] Tanner A.C., Wu C.C. (1992). Adaptation of the Sensititre broth microdilution technique to antimicrobial susceptibility testing of Mycoplasma gallisepticum. Avian Dis..

[20] Hannan P.C., O’Hanlon P.J., Rogers N.H. (1989). In vitro evaluation of various quinolone antibacterial agents against veterinary mycoplasmas and porcine respiratory bacterial pathogens. Res. Vet. Sci..

[21] Li Y., Feng B., Gu X., Yang D., Zeng Z., Zhang B., Ding H. (2016). Correlation of PK/PD Indices with Resistance Selection for Cefquinome againstStaphylococcus aureusin anIn VitroModel. Front. Microbiol..

[22] Marois C., Gottschalk M., Morvan H., Fablet C., Madec F., Kobisch M. (2009). Experimental infection of SPF pigs with Actinobacillus pleuropneumoniae serotype 9 alone or in association with Mycoplasma hyopneumoniae. Vet. Microbiol..

[23] Beier L.S., Siqueira F.M., Schrank I.S. (2018). Evaluation of growth and gene expression of Mycoplasma hyopneumoniae and Mycoplasma hyorhinis in defined medium. Mol. Biol. Rep..

[24] Maes D., Sibila M., Kuhnert P., Segales J., Haesebrouck F., Pieters M. (2018). Update on Mycoplasma hyopneumoniae infections in pigs: Knowledge gaps for improved disease control. Transbound. Emerg. Dis..

[25] Tam V.H., Schilling A.N., Vo G., Kabbara S., Kwa A.L., Wiederhold N.P., Lewis R.E. (2005). Pharmacodynamics of polymyxin B against Pseudomonas aeruginosa. Antimicrob. Agents Chemother..

[26] Nan Z., Gu X., Ye X., Xun W., Zhang B., Zhang L., Shen X., Jiang H., Ding H. (2016). The PK/PD Interactions of Doxycycline againstMycoplasma gallisepticum. Front. Microbiol..

[27] Vicca J., Stakenborg T., Maes D., Butaye P., Peeters J., de Kruif A., Haesebrouck F. (2004). In vitro susceptibilities of Mycoplasma hyopneumoniae field isolates. Antimicrob. Agents Chemother..

[28] Felde O., Kreizinger Z., Sulyok K.M., Hrivnak V., Kiss K., Jerzsele A., Biksi I., Gyuranecz M. (2018). Antibiotic susceptibility testing of Mycoplasma hyopneumoniae field isolates from Central Europe for fifteen antibiotics by microbroth dilution method. PLoS ONE.

[29] Hannan P.C. (2000). Guidelines and recommendations for antimicrobial minimum inhibitory concentration (MIC) testing against veterinary mycoplasma species. International Research Programme on Comparative Mycoplasmology. Vet. Res..

[30] Whithear K.G., Bowtell D.D., Ghiocas E., Hughes K.L. (1983). Evaluation and use of a micro-broth dilution procedure for testing sensitivity of fermentative avian mycoplasmas to antibiotics. Avian Dis..

[31] Drlica K., Zhao X. (2007). Mutant selection window hypothesis updated. Clin. Infect. Dis..

[32] Fran Oise V.B., Stéphane C., Cristina S., Hugues C., Donatienne T., Marie-Paule M.L., Tulkens P.M. (2004). Cellular pharmacokinetics and pharmacodynamics of the glycopeptide antibiotic oritavancin (LY333328) in a model of J774 mouse macrophages. Antimicrob. Agents Chemother..

[33] Carbon C. (1998). Pharmacodynamics of macrolides, azalides, and streptogramins: Effect on extracellular pathogens. Clin. Infect. Dis..

[34] Van B.F., Tulkens P.M. (2001). Macrolides: Pharmacokinetics and pharmacodynamics. Int. J. Antimicrob. Agents.

[35] Tam V.H., Kabbara S., Vo G., Schilling A.N., Coyle E.A. (2006). Comparative pharmacodynamics of gentamicin against Staphylococcus aureus and Pseudomonas aeruginosa. Antimicrob. Agents Chemother..

[36] Zhou Y.F., Peng H.M., Bu M.X., Liu Y.H., Sun J., Liao X.P. (2017). Pharmacodynamic Evaluation and PK/PD-Based Dose Prediction of Tulathromycin: A Potential New Indication for Streptococcus suis Infection. Front. Pharmacol..

[37] Zhao Y., Guo L.L., Fang B., Liu B. (2018). Pharmacokinetic/pharmacodynamic (PK/PD) evaluation of tulathromycin against Haemophilus parasuis in an experimental neutropenic guinea pig model. PLoS ONE.

[38] Toutain P.L., Potter T., Pelligand L., Lacroix M., Illambas J., Lees P. (2017). Standard PK/PD concepts can be applied to determine a dosage regimen for a macrolide: The case of tulathromycin in the calf. J. Vet. Pharmacol. Ther..

[39] Zhou Q., Zhang G., Wang Q., Liu W., Huang Y., Yu P., Li Y., Ding H., Fang B. (2017). Pharmacokinetic/Pharmacodynamic Modeling of Tulathromycin against Pasteurella multocida in a Porcine Tissue Cage Model. Front. Pharmacol..

